# Sunday Dinners

**Published:** 1856-02

**Authors:** 


### SUNDAY DINNERS.

We are not quite sure but that the *Presbyterians* are, after all, nearer right, all things considered, than any other denomination of Christians. There is a vein of sturdy propriety, of sterling philosophy, running through their commonest customs, well worthy of a note—we speak more particularly of the bona fide, the real, identical, original, old-fashioned sort of *Pris-by-churns*, as it is pronounced, away off yonder in the woods of the West, where we sprouted and came up, and have been upping ever since, until *Medicated Inhalation* came along, took the wind out of our sails, cured up all the consumptives, and left us nothing to do but stand with gaping mouth and open eyes, wondering where we will *fetch up*, cogitating *whereunto these things will grow*—alternating with the more agreeable pastime, of rolling over the floor with our little ALICE, who grows sweeter every day, as with the weight of a baker's dozen of months on her head, she is taking her first lesson in pedestrianation—read carefully, it is not predestination we are talking about, although speaking of *Presbyterians*, we are next door to it. Can a heart be ever more full of pure delight, than when looking at one's own child's desperate first efforts at walking; how it hesitates, half moves a foot, then hesitates again; then catching a glance from its mother's eyes and beckoning finger, it summons up all its resolution, and with open mouth and upraised hands, makes the desperate plunge, and with half a step, throws itself foward in its mother's arms, never doubting that she would let it fall; by degrees, a step or two more are taken, until at length the wonderful feat is performed, of toddling clear across the room, and you read the triumph in its eyes, as legibly as if a con queror had won a world.

As we were saying, *Sunday Dinners!*—that is, a "Presbyterian Dinner," as it is denominated on the other side of the *Alleghenies*, is most philosophical; it is a *cold* dinner, and its philosophy consists—

1st. In its piety.

2d. In its humanity.

3d. In its prophylactiveness.

Its *piety* is evident, from its allowing more time for that religious reflection, which becomes the Sabbath day; and which, being *cumbered with much serving*, as effectually prevents, as it did in MARTHA'S time.

That it is *humane* to have as little cooking done on Sunday, and thus giving as much *rest* to our servants as practicable, no one will deny.

As to the *healthfulness* of a cold dinner on Sundays, a moment's reflection will be conclusive.

As we take very much less exercise on the Sabbath day than when engaged in our ordinary avocations, we need that much less food. No one can eat as much of a cold dinner as he would if it were smoking hot. There is no danger of our not eating enough dinner on Sundays, let it be ever so cold and uninviting; for if any business man would take nothing at all for his Sunday dinner, and for the following supper were to drink a single cup of any kind of tea, weak and hot, and eat with it a bit of toast or a piece of cold bread and butter, he would be all the better for it in mind and body next day; and would go to his business on a Monday morning, with a vigor and an elasticity which that man never knows who makes his Sunday dinner *the* dinner of the week.

Taking so much less exercise on Sundays than on a week-day, and stimulated to eat more on that day by its superior excellency, aided by idleness, there is of necessity a repletion, an over-supply of food, which will be as certainly disastrous, as the feeding of a locomotive with more fuel while she is standing still, than when she is going ahead, with her long retinue of passengers and freight.

But in a sober, religious point of view, those inviting Sunday dinners are not judicious; the nervous energy is drawn to the stomach in extreme quantities, in order to dispose of the overload, leaving the brain scantily supplied, causing dulness, drowsiness, and almost stupidity, wholly unfitting the mind for proper attention to the religious exercises of the afternoon, the palpable cause of wasted sermons, of wasted opportunities. This subject is worth a serious thought on the part of pious people, especially those who have a growing family. Cold bread and meat, with pie or baked apples, and a single cup of good hot tea or coffee, make a good enough Sunday dinner for anybody.

